# Supplementary material for: Super-resolution mapping in rod photoreceptors identifies rhodopsin trafficking through the inner segment plasma membrane as an essential subcellular pathway
Source: PLoS Biol. 2024 Jan 8;22(1):e3002467. doi: 10.1371/journal.pbio.3002467 (PMC10773939; doi:10.1371/journal.pbio.3002467)
Supplement: S1 Fig — (A, B) Western blot test of NbGFP-A647 immunolabeling specificity. (A) WT and Rho-GFP/+ retinal lysates used as either 0.5% or 2% total volume of 1 mouse retina. In Rho-GFP/+ lysates, a prominent and specific NbGFP+ band was found approximately 60 kDa corresponding to monomeric Rho-GFP protein. Both WT and Rho-GFP/+ lysates contained Rho-C-1D4-positive bands corresponding to endogenous mouse Rho protein. Mouse Rho protein levels are apparently lower in Rho-GFP/+ lysates—corresponding to the one WT Rho allele in these mice. In addition, Rho-C-1D4 antibody does not immunolabel Rho-GFP protein from Rho-GFP/+ lysates. IB, immunoblot condition. (B) WT and Rho-GFP-1D4/+ retinal lysates used as 2% total volume of 1 mouse retina. In Rho-GFP-1D4/+ lysates specific NbGFP-A647+ and Rho-C-1D4+ bands are found approximately 60 kDa corresponding to monomeric Rho-GFP-1D4 protein. As is (A), endogenous mouse Rho protein levels are lower in Rho-GFP-1D4/+ lysates. For all western blots, molecular weight marker sizes are indicated in kDa. (C) To test NbGFP-A647 immunolabeling specificity with immunofluorescence, 10 μm eye cup cryosections from Rho-GFP/+ mice (age P71) and from WT mice (age P60) were immunolabeled with NbGFP-A647 and counterstained with DAPI. NbGFP-647 specifically labels the OSs in the Rho-GFP/+ section (magenta) and overlaps with GFP (green). No detectable NbGFP-A647 fluorescence was detected in the WT sections. Scale bar values match adjacent panels when not given. (D) Rho-GFP/+ or (E) Rho-GFP-1D4/+ adult retinal lysates treated with either Protein Deglycosylation Mix II from NEB (containing PNGase F) or buffer only. After treatment, 5 μg of total protein was loaded for SDS-PAGE, transfer, and primary antibody labeling. A shift to lower molecular weight was observed for all Rho bands, including Rho-GFP bands from Rho-GFP/+ treated lysates probed with NbGFP-A647. In (D), Rho-GFP was also detected with Rho-N-4D2 immunolabeling; monomeric Rho-GFP bands are indicated wi [file pbio.3002467.s001.pdf]

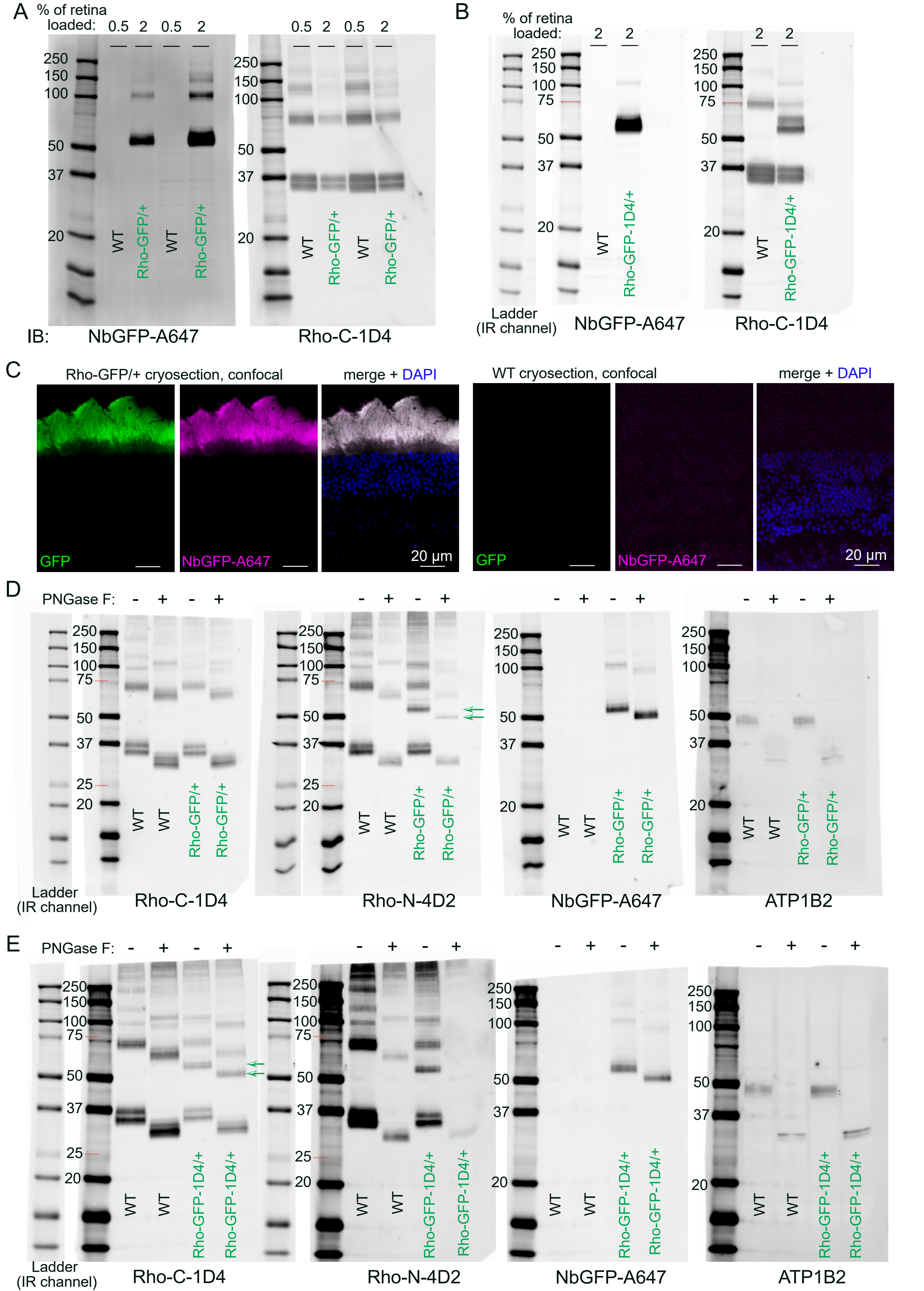

A
% of retina loaded:
0.5
2
0.5
2
250
150
100
50
37
20
WT
Rho-GFP/+
IB:
NbGFP-A647
Rho-C-1D4
B
Ladder (IR channel)
NbGFP-A647
Rho-C-1D4
Rho-GFP-1D4/+
75
C
Rho-GFP/+ cryosection, confocal
merge + DAPI
GFP
NbGFP-A647
20 µm
WT cryosection, confocal
D
PNGase F:
-
+
25
Rho-N-4D2
ATP1B2
E

Figure S1. (A, B) Western blot test of NbGFP-A647 immunolabeling specificity. (A) WT and Rho-GFP/+ retinal lysates used as either 0.5% or 2% total volume of 1 mouse retina. In Rho-GFP/+ lysates, a prominent and specific NbGFP+ band was found ~60 kDa corresponding to monomeric Rho-GFP protein. Both WT and Rho-GFP/+ lysates contained Rho-C-1D4-positive bands corresponding to endogenous mouse Rho protein. Mouse Rho protein levels are apparently lower in Rho-GFP/+ lysates – corresponding to the one WT Rho allele in these mice. In addition, Rho-C-1D4 antibody does not immunolabel Rho-GFP protein from Rho-GFP/+ lysates. IB=immunoblot condition. (B) WT and Rho-GFP-1D4/+ retinal lysates used as 2% total volume of 1 mouse retina. In Rho-GFP-1D4/+ lysates specific NbGFP-A647+ and Rho-C-1D4+ bands are found ~60 kDa corresponding to monomeric Rho-GFP-1D4 protein. As is (A), endogenous mouse Rho protein levels are lower in Rho-GFP-1D4/+ lysates. For all western blots, molecular weight marker sizes are indicated in kDa. (C) To test NbGFP-A647 immunolabeling specificity with immunofluorescence, 10 µm eye cup cryosections from Rho-GFP/+ mice (age P71) and from WT mice (age P60) were immunolabeled with NbGFP-A647 and counterstained with DAPI. NbGFP-647 specifically labels the outer segments (OS) in the Rho-GFP/+ section (magenta) and overlaps with GFP (green). No detectable NbGFP-A647 fluorescence was detected in the WT sections. Scalebar values match adjacent panels when not given. (D) Rho-GFP/+ or (E) Rho-GFP-1D4/+ adult retinal lysates treated with either Protein Deglycosylation Mix II from NEB (containing PNGase F) or buffer only. After treatment, 5 µg of total protein was loaded for SDS-PAGE, transfer and primary antibody labeling. A shift to lower molecular weight was observed for all Rho bands, including Rho-GFP bands from Rho-GFP/+ treated lysates probed with NbGFP-A647. In (D) Rho-GFP was also detected with Rho-N-4D2 immunolabeling; monomeric Rho-GFP bands are indicated with green arrows on this blot. In (E) Rho-GFP-1D4 was detected with Rho-C-1D4 immunolabeling and monomeric Rho-GFP-1D4 bands are indicated with green arrows.
