# Supplementary material for: Super-resolution mapping in rod photoreceptors identifies rhodopsin trafficking through the inner segment plasma membrane as an essential subcellular pathway
Source: PLoS Biol. 2024 Jan 8;22(1):e3002467. doi: 10.1371/journal.pbio.3002467 (PMC10773939; doi:10.1371/journal.pbio.3002467)
Supplement: S2 Fig — (A) TEM images of Rho-GFP/+ retina slices that were either unpeeled, peeled 4 times, or peeled 8 times (the IS-enriched condition). Images were pseudocolored to point out key rod structures as follows: OS = yellow, IS = magenta, CCs/BBs = blue. Scale bar values match adjacent panels when not labeled. (B) Alternate TEM single rod examples from Rho-GFP/+ IS-enriched retinas. The IS plasma membrane is annotated with magenta arrows. (C) SIM images from IS-enriched Rho-GFP/+ retinas immunolabeled for NbGFP-A647 (magenta), STX3 (cyan), and centrin (yellow). To demonstrate Rho-GFP colocalization with STX3 at the IS plasma membrane, row average intensity plots are shown for portions of the IS from 2 different magnified single rod examples marked with a dashed line. (D, E) Additional single rod SIM z-projection images of WT IS-enriched retina sections immunolabeled with either (D) Rho-C-1D4 (magenta) or (E) Rho-N-4D2 (magenta); both co-immunolabeled with STX3 (cyan) and centrin-2 (yellow) antibodies. White arrows indicate Rho fluorescence that is colocalized with STX3 at the plasma membrane. Numerical values corresponding to all graphical data are provided in Table F in S1 Data. BB, basal body; CC, connecting cilium; IS, inner segment; NbGFP-A647, GFP nanobody Alexa 647 conjugate; OS, outer segment; Rho, rhodopsin; SIM, structured illumination microscopy; STX3, syntaxin 3; TEM, transmission electron microscopy; WT, wild-type. (PDF) [file pbio.3002467.s002.pdf]

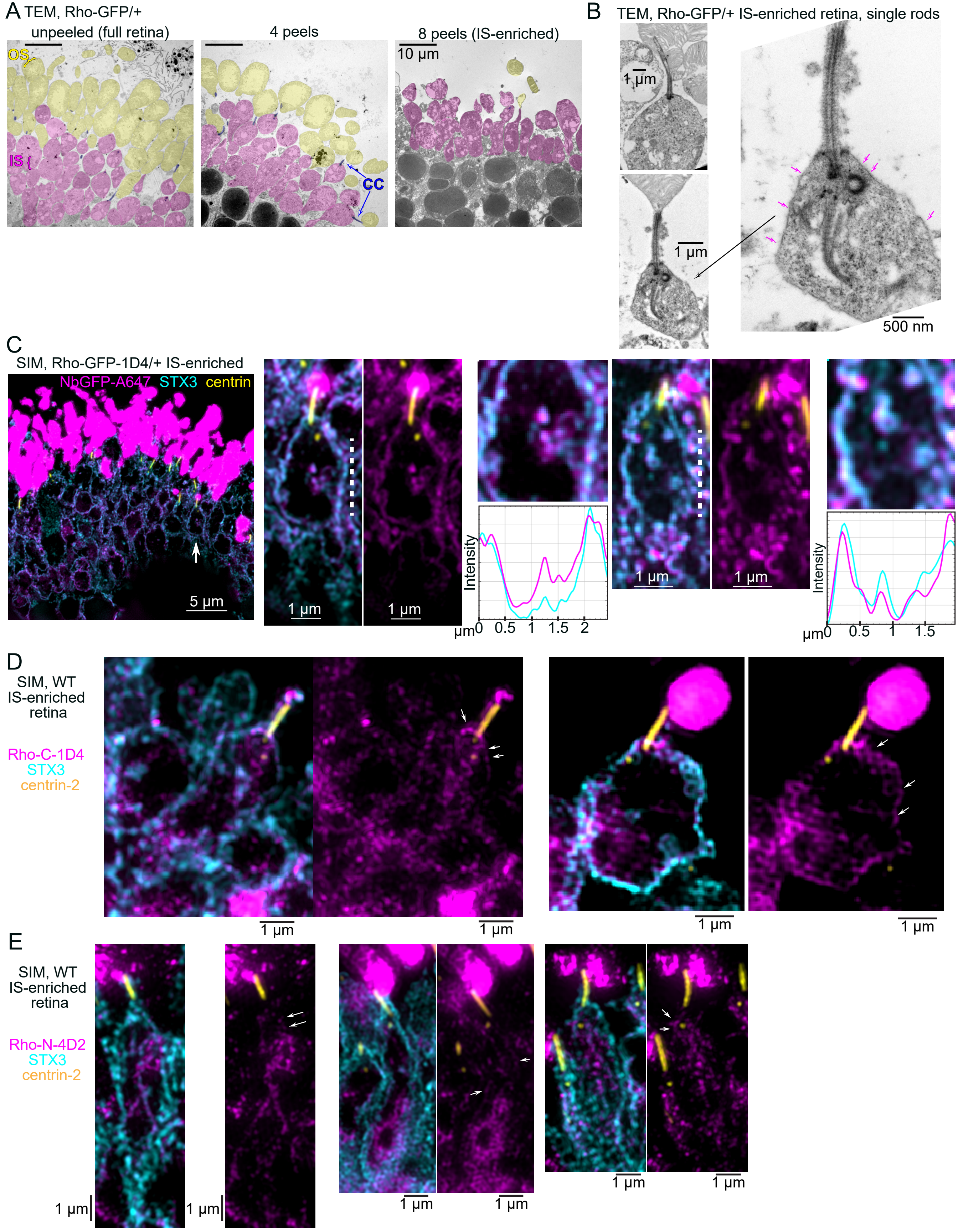

A TEM, Rho-GFP/+
unpeeled (full retina)
4 peels
8 peels (IS-enriched)
10 µm
OS
IS
CC
B TEM, Rho-GFP/+ IS-enriched retina, single rods
1 µm
1 µm
500 nm
C
SIM, Rho-GFP-1D4/+ IS-enriched
NbGFP-A647 STX3 centrin
5 µm
1 µm
1 µm
Intensity
µm 0 0.5 1 1.5 2
1 µm
1 µm
Intensity
µm 0 0.5 1 1.5
D
SIM, WT IS-enriched retina
Rho-C-1D4
STX3
centrin-2
1 µm
1 µm
1 µm
1 µm
E
SIM, WT IS-enriched retina
Rho-N-4D2
STX3
centrin-2
1 µm
1 µm
1 µm
1 µm
1 µm
1 µm

Figure S2. (A) TEM images of Rho-GFP/+ retina slices that were either unpeeled, peeled 4 times or peeled 8 times (the IS-enriched condition). Images were pseudocolored to point out key rod structures as follows: OS = yellow, IS = magenta, connecting cilia (CC)/basal bodies = blue. Scalebar values match adjacent panels when not labeled. (B) Alternate TEM single rod examples from Rho-GFP/+ IS-enriched retinas. The IS plasma membrane is annotated with magenta arrows. (C) SIM images from IS-enriched Rho-GFP/+ retinas immunolabeled for NbGFP-A647 (magenta), STX3 (cyan), and centrin (yellow). To demonstrate Rho-GFP colocalization with STX3 at the IS plasma membrane, row average intensity plots are shown for portions of the IS from 2 different magnified single rod examples marked with a dashed line. (D, E) Additional single rod SIM z-projection images of WT IS-enriched retina sections immunolabeled with either (D) Rho-C-1D4 (magenta) or (E) Rho-N-4D2 (magenta); both co-immunolabeled with STX3 (cyan) and centrin-2 (yellow) antibodies. White arrows indicate Rho fluorescence that is colocalized with STX3 at the plasma membrane. Numerical values corresponding to all graphical data are provided in Table F in S1 Data.
