# Supplementary material for: Super-resolution mapping in rod photoreceptors identifies rhodopsin trafficking through the inner segment plasma membrane as an essential subcellular pathway
Source: PLoS Biol. 2024 Jan 8;22(1):e3002467. doi: 10.1371/journal.pbio.3002467 (PMC10773939; doi:10.1371/journal.pbio.3002467)
Supplement: S3 Fig — Alternate single rod STORM reconstruction examples from (A) Rho-GFP staining conditions colabeled with STX3 (cyan) and centrin-2 yellow. Each example features plots of STORM molecule coordinates within the STX3+ IS hull for each channel and an adjacent plot with randomly plotted molecules within the IS hull (orange), as well as frequency and CDF graphs for distance to hull measurements from the plotted STORM coordinates. Molecule counts: (Ai) Rho-GFP n = 9,634, STX3 n = 7,643, Random n = 9,634; (Aii) Rho-GFP n = 4,298, STX3 n = 14,385, Random n = 4,298; (Aiii) Rho-GFP n = 4,709, STX3 n = 10,385, Random n = 4,709. (B) STORM reconstructions of IS-enriched Rho-GFP-1D4/+ retina sections immunolabeled with NbGFP-A647 (cyan) and STX3 (cyan) and centrin-2 (yellow) antibodies. OSs are indicated. The centrin-2+ widefield images are superimposed on STORM reconstruction images. In single rod examples, the IS region is indicated, and the IS hull is outlined in cyan in a duplicate image. For each example Rho-GFP-1D4 and STX3 STORM molecule coordinates within the IS hull are plotted (top example: Rho-GFP-1D4 molecules = 8,135, STX3 molecules = 24,826; bottom example: Rho-GFP-1D4 molecules = 3,326, STX3 molecules = 17,765). In the adjacent plot, a random distribution of coordinates within the IS hull matching the number of Rho-GFP-1D4 molecules (4,239) are plotted in orange. Nearest distance-to-hull measurements for Rho-GFP-1D4, STX3, and random molecules are plotted in a frequency and CDF graphs. Colors in the graphs match the molecule plots. (C-F) Alternate single rod STORM reconstruction examples from (C) Rho-C-1D4, (D) Rho-N-4D2, (E) SNAP25, and (F) PDC conditions all colabeled with STX3 (cyan) and centrin-2 (yellow) antibodies. Molecule counts (Ci) Rho-C-1D4 n = 1,255, STX3 n = 60,036, Random n = 1,255; (Cii) Rho-C-1D4 n = 9,586, STX3 n = 37,569, Random n = 9,586; (Ciii) Rho-C-1D4 n = 1,152, STX3 n = 66,161, Random n = 1,152; (Di) Rho-N-4D2 n = 4,965; STX3 n = 67,836; Random [file pbio.3002467.s003.pdf]

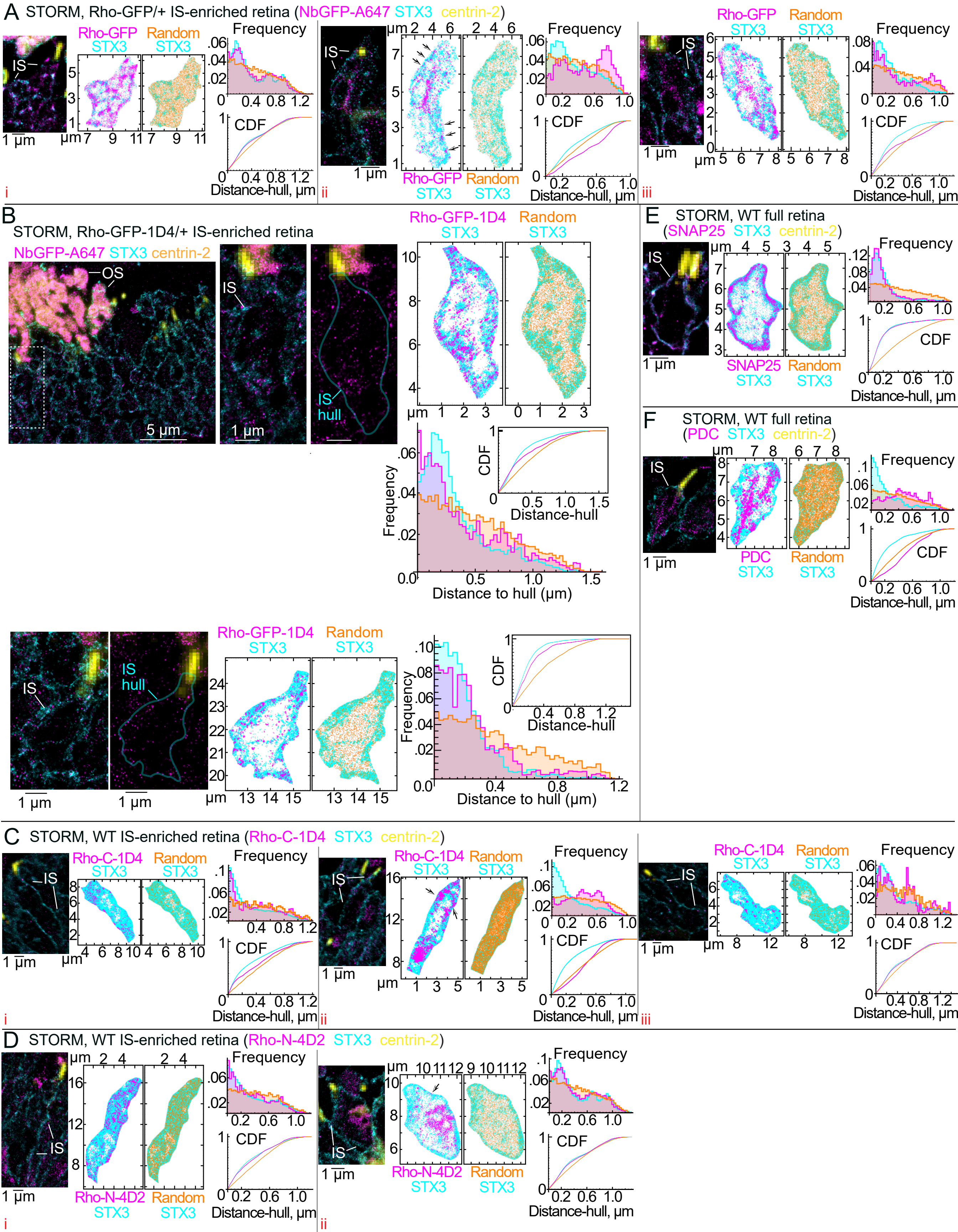
A STORM, Rho-GFP/+ IS-enriched retina (NbGFP-A647 STX3 centrin-2)
IS
1 µm
Rho-GFP STX3
Random STX3
Frequency
CDF
Distance-hull, µm
i
ii
iii
B
STORM, Rho-GFP-1D4/+ IS-enriched retina
NbGFP-A647 STX3 centrin-2
OS
5 µm
1 µm
IS hull
Rho-GFP-1D4 STX3
Random STX3
Frequency
Distance to hull (µm)
CDF
Distance-hull
E STORM, WT full retina (SNAP25 STX3 centrin-2)
SNAP25 STX3
Random STX3
F STORM, WT full retina (PDC STX3 centrin-2)
PDC STX3
Random STX3
C STORM, WT IS-enriched retina (Rho-C-1D4 STX3 centrin-2)
Rho-C-1D4 STX3
Random STX3
D STORM, WT IS-enriched retina (Rho-N-4D2 STX3 centrin-2)
Rho-N-4D2 STX3
Random STX3

Figure S3. Alternate single rod STORM reconstruction examples from (A) Rho-GFP staining conditions co-labeled with STX3 (cyan) and centrin-2 yellow. Each example features plots of STORM molecule coordinates within the STX3+ IS hull for each channel and an adjacent plot with randomly plotted molecules within the IS hull (orange), as well as frequency and CDF graphs for distance to hull measurements from the plotted STORM coordinates. Molecule counts: (Ai) Rho-GFP n=9,634, STX3 n=7,643, Random n=9,634; (Aii) Rho-GFP n=4,298, STX3 n=14,385, Random n=4,298; (Aiii) Rho-GFP n=4,709, STX3 n=10,385, Random n=4,709. (B) STORM reconstructions of IS-enriched Rho-GFP-1D4/+ retina sections immunolabeled with NbGFP-A647 (cyan) and STX3 (cyan) and centrin-2 (yellow) antibodies. OSs are indicated. The centrin-2+ widefield images are superimposed on STORM reconstruction images. In single rod examples, the IS region is indicated, and the IS hull is outlined in cyan in a duplicate image. For each example Rho-GFP-1D4 and STX3 STORM molecule coordinates within the IS hull are plotted (top example: Rho-GFP-1D4 molecules = 8,135, STX3 molecules = 24,826; bottom example: Rho-GFP-1D4 molecules = 3,326, STX3 molecules = 17,765). In the adjacent plot, a random distribution of coordinates within the IS hull matching the number of Rho-GFP-1D4 molecules (4,239) are plotted in orange. Nearest distance to hull measurements for Rho-GFP-1D4, STX3 and random molecules are plotted in a frequency and CDF graphs. Colors in the graphs match the molecule plots. (C-F) Alternate single rod STORM reconstruction examples from (C) Rho-C-1D4, (D) Rho-N-4D2, (E) SNAP25 and (F) PDC conditions all co-labeled with STX3 (cyan) and centrin-2 (yellow) antibodies. Molecule counts (Ci) Rho-C-1D4 n=1,255, STX3 n=60,036, Random n=1,255; (Cii) Rho-C-1D4 n=9,586, STX3 n=37,569, Random n=9,586; (Ciii) Rho-C-1D4 n=1,152, STX3 n=66,161, Random n=1,152; (Di) Rho-N-4D2 n=4,965; STX3 n=67,836; Random n=4,965; (Dii) Rho-N-4D2 n=4,975; STX3 n=13,387; Random n=4,975; (E) SNAP25 n=7,697; STX3 n=25,196; Random n=7,697 (F) PDC n=10,920; STX3 n=18,619, Random n=10,920. Black arrows = Rho STORM molecules located at the STX3+ IS hull in rod examples where the majority of Rho molecules are internal (Aii, Cii, Dii). Numerical values corresponding to all graphical data are provided in Table G in S1 Data.
