# Supplementary material for: Super-resolution mapping in rod photoreceptors identifies rhodopsin trafficking through the inner segment plasma membrane as an essential subcellular pathway
Source: PLoS Biol. 2024 Jan 8;22(1):e3002467. doi: 10.1371/journal.pbio.3002467 (PMC10773939; doi:10.1371/journal.pbio.3002467)
Supplement: S4 Fig — (A, B) Violin plot graphs of STORM distance to hull normalized frequency values within (A) 0.1 μm and (B) 0.3 μm. N values are the same as in Fig 6F. Comparisons were tested for statistical significance using the Mann–Whitney U test. (A) PDC vs. Rho-GFP **P value = 0.0011; PDC vs. Rho-GFP-1D4 ***P value < 0.00001; PDC vs. 1D4 ***P = 0.0003; PDC vs. 4D2 *P = 0.0172. (B) PDC vs. Rho-GFP ***P value = 0.0003; PDC vs. Rho-GFP-1D4 ***P value < 0.00001; PDC vs. 1D4 **P = 0.0033. (C, D) Immunogold localization of STX3 and Rho in mouse rods. (C) Single rod IS electron micrograph examples from a WT mouse retinas immunolabeled with STX3 antibody and nanogold secondary antibody. In a threshold image showing only the STX3+ immunogold particles, the approximate location of the IS plasma membrane is outlined in cyan. The outline is also continuous with the CC membrane. (D) Electron micrograph examples of rod ISs from IS-enriched WT mouse retinas immunolabeled with Rho-C-1D4. The IS plasma membrane is outlined in cyan in the threshold image. Some non-punctate staining from the BB and CC axoneme is present in the threshold images. mito = mitochondria. Numerical values corresponding to all graphical data are provided in Table H in S1 Data. BB, basal body; CC, connecting cilium; IS, inner segment; PDC, phosducin; Rho, rhodopsin; STORM, stochastic optical reconstruction microscopy; STX3, syntaxin 3; WT, wild-type. (PDF) [file pbio.3002467.s004.pdf]

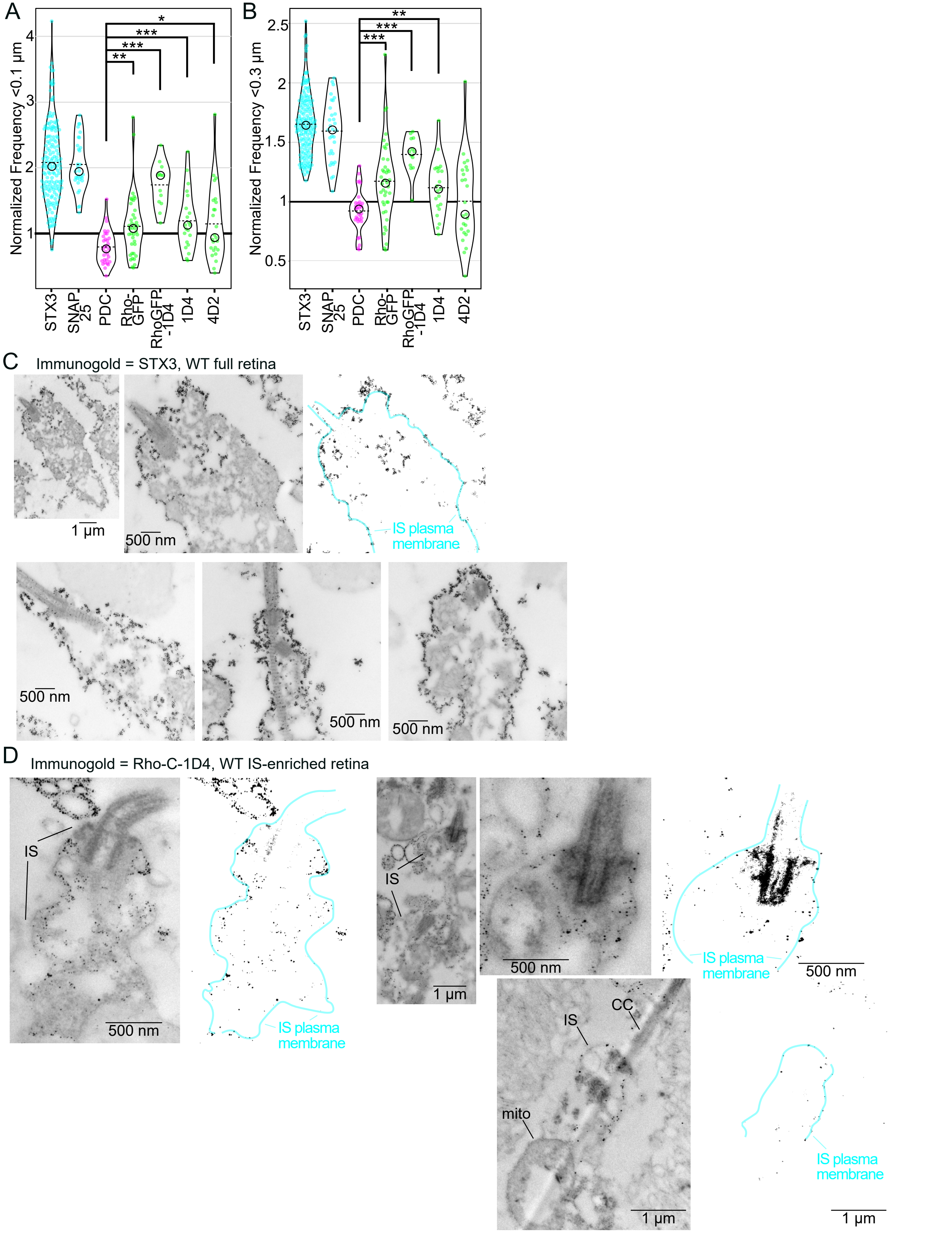
A
Normalized Frequency <0.1 µm
*
***
***
**
STX3
SNAP 25
PDC
Rho-GFP
RhoGFP -1D4
1D4
4D2
B
Normalized Frequency <0.3 µm
**
***
***
STX3
SNAP 25
PDC
Rho-GFP
RhoGFP -1D4
1D4
4D2
C
Immunogold = STX3, WT full retina
1 µm
500 nm
IS plasma membrane
500 nm
500 nm
500 nm
D
Immunogold = Rho-C-1D4, WT IS-enriched retina
IS
500 nm
IS plasma membrane
IS
1 µm
500 nm
IS plasma membrane
500 nm
CC
IS
mito
1 µm
IS plasma membrane
1 µm

Figure S4. (A, B) Violin plot graphs of STORM distance to hull normalized frequency values within (A) 0.1 µm and (B) 0.3 µm. N values are the same as in Figure 6F. Comparisons were tested for statistical significance using the Mann-Whitney U test. (A) PDC vs Rho-GFP **P-value = 0.0011; PDC vs Rho-GFP-1D4 ***P-value < 0.00001; PDC vs 1D4 ***P=0.0003; PDC vs 4D2 *P= 0.0172. (B) PDC vs Rho-GFP ***P-value = 0.0003; PDC vs Rho-GFP-1D4 ***P-value < 0.00001; PDC vs 1D4 **P= 0.0033. (C,D) Immunogold localization of syntaxin 3 and rhodopsin in mouse rods. (C) Single rod inner segment electron micrograph examples from a WT mouse retinas immunolabeled with STX3 antibody and nanogold secondary antibody. In a threshold image showing only the STX3+ immunogold particles, the approximate location of the IS plasma membrane is outlined in cyan. The outline is also continuous with the CC membrane. (D) Electron micrograph examples of rod ISs from IS-enriched WT mouse retinas immunolabeled with Rho-C-1D4. The IS plasma membrane is outlined in cyan in the threshold image. Some non-punctate staining from the BB and CC axoneme is present in the threshold images. mito = mitochondria. Numerical values corresponding to all graphical data are provided in Table H in S1 Data.
