# Supplementary material for: Super-resolution mapping in rod photoreceptors identifies rhodopsin trafficking through the inner segment plasma membrane as an essential subcellular pathway
Source: PLoS Biol. 2024 Jan 8;22(1):e3002467. doi: 10.1371/journal.pbio.3002467 (PMC10773939; doi:10.1371/journal.pbio.3002467)
Supplement: S5 Fig — (A) Alternate STORM single rod examples from Rho-GFP/+ IS-enriched retinas immunolabeled with NbGFP-A647 (magenta), and centrin and STX3 antibodies (combined, yellow), and Rab11a. (B) STORM examples for Rho-GFP-1D4/+ IS-enriched retinas immunolabeled with NbGFP-A647 (magenta), and centrin and STX3 antibodies (combined, yellow), and Rab11a. Rab11a+ clusters identified with Voronoi tessellation are in white. (C, D) STORM examples for Rho-GFP-1D4/+ IS-enriched retinas immunolabeled with NbGFP-A647 (magenta), and centrin and STX3 antibodies (combined, yellow), and either (C) DYNC1H1 or (D) Rootletin antibody. (E) Diagram of the mouse rod IS architecture and the localization pattern of Rho. IS, inner segment; NbGFP-A647, GFP nanobody Alexa 647 conjugate; Rho, rhodopsin; STORM, stochastic optical reconstruction microscopy; STX3, syntaxin 3. (PDF) [file pbio.3002467.s005.pdf]

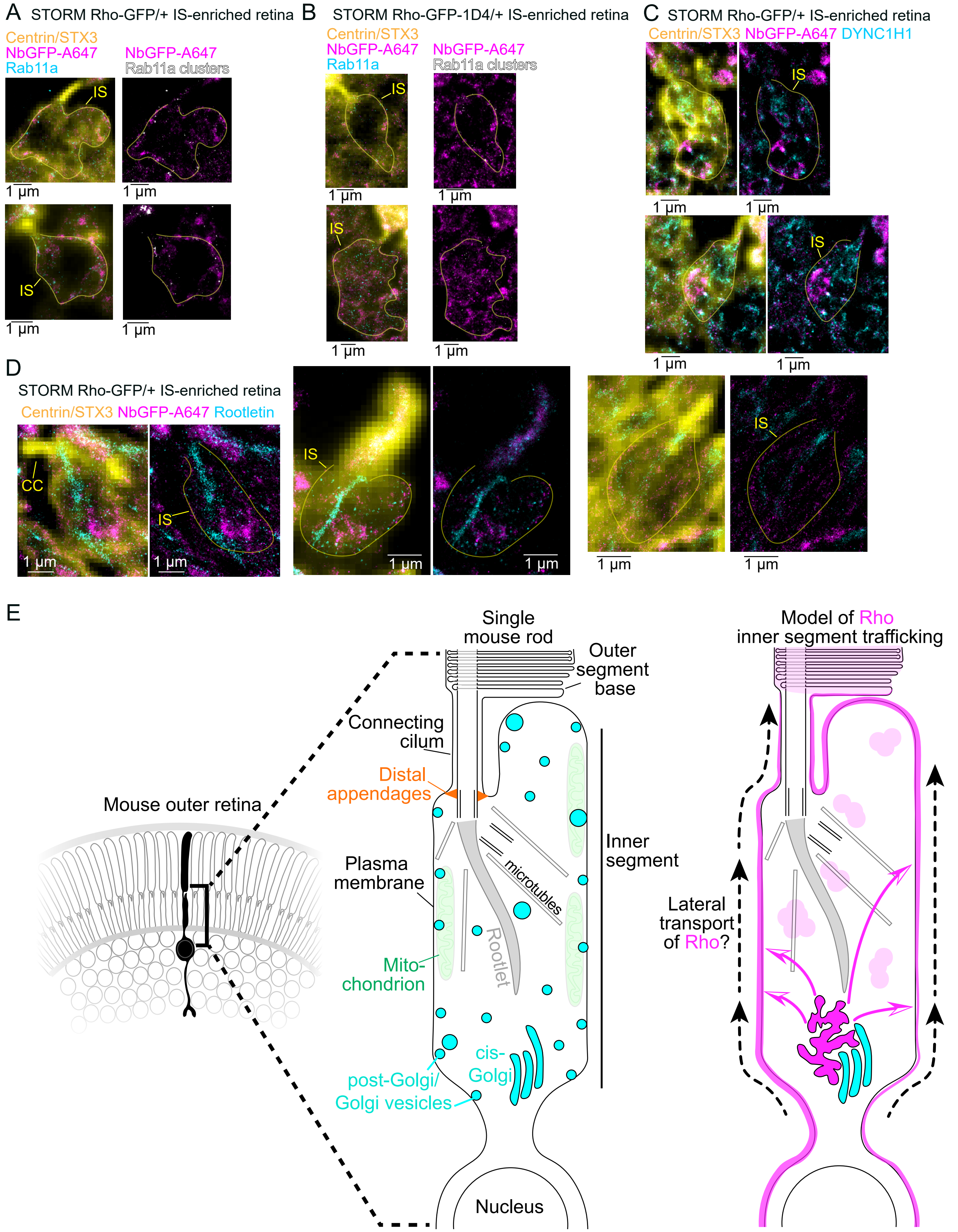

A STORM Rho-GFP/+ IS-enriched retina
Centrin/STX3
NbGFP-A647
Rab11a
NbGFP-A647
Rab11a clusters
IS
1 µm
B STORM Rho-GFP-1D4/+ IS-enriched retina
C STORM Rho-GFP/+ IS-enriched retina
Centrin/STX3 NbGFP-A647 DYNC1H1
D
STORM Rho-GFP/+ IS-enriched retina
Centrin/STX3 NbGFP-A647 Rootletin
CC
E
Mouse outer retina
Single mouse rod
Outer segment base
Connecting cilum
Distal appendages
Plasma membrane
microtubles
Rootlet
Mito-chondrion
cis-Golgi
post-Golgi/ Golgi vesicles
Inner segment
Nucleus
Model of Rho inner segment trafficking
Lateral transport of Rho?

Figure S5. (A) Alternate STORM single rod examples from Rho-GFP/+ IS-enriched retinas immunolabeled with NbGFP-A647 (magenta), and centrin and STX3 antibodies (combined, yellow), and Rab11a. (B) STORM examples for Rho-GFP-1D4/+ IS-enriched retinas immunolabeled with NbGFP-A647 (magenta), and centrin and STX3 antibodies (combined, yellow), and Rab11a. Rab11a+ clusters identified with Voronoi tessellation are in white. (C-D) STORM examples for Rho-GFP-1D4/+ IS-enriched retinas immunolabeled with NbGFP-A647 (magenta), and centrin and STX3 antibodies (combined, yellow), and either (C) DYNC1H1 or (D) Rootletin antibody. (E) Diagram of the mouse rod inner segment architecture and the localization pattern of rhodopsin.
