# Supplementary figures and images for: Super-resolution mapping in rod photoreceptors identifies rhodopsin trafficking through the inner segment plasma membrane as an essential subcellular pathway
Source: PLoS Biol. 2024 Jan 8;22(1):e3002467. doi: 10.1371/journal.pbio.3002467 (PMC10773939; doi:10.1371/journal.pbio.3002467)

Source Data for Figure 2

B

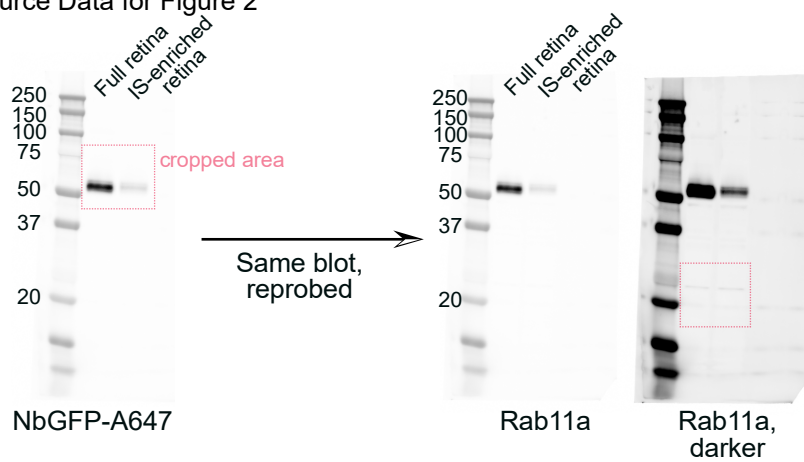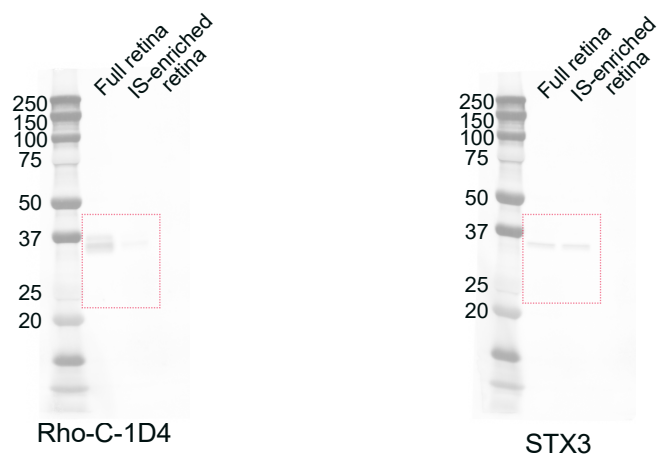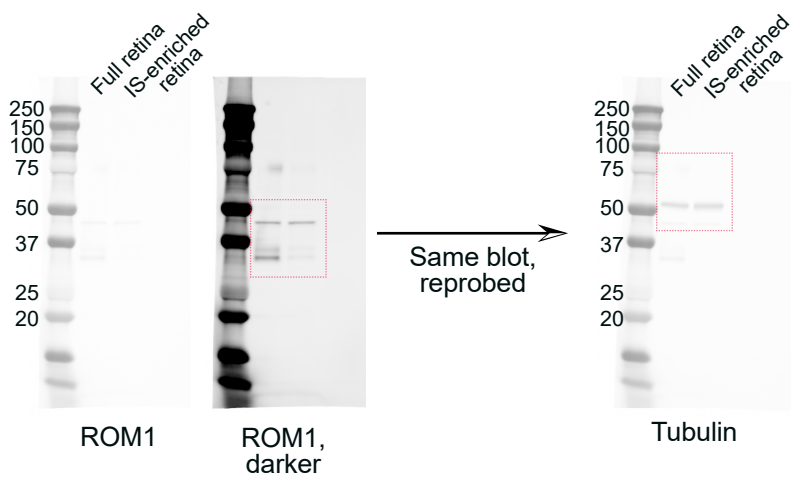

# Source Data for Figure 8

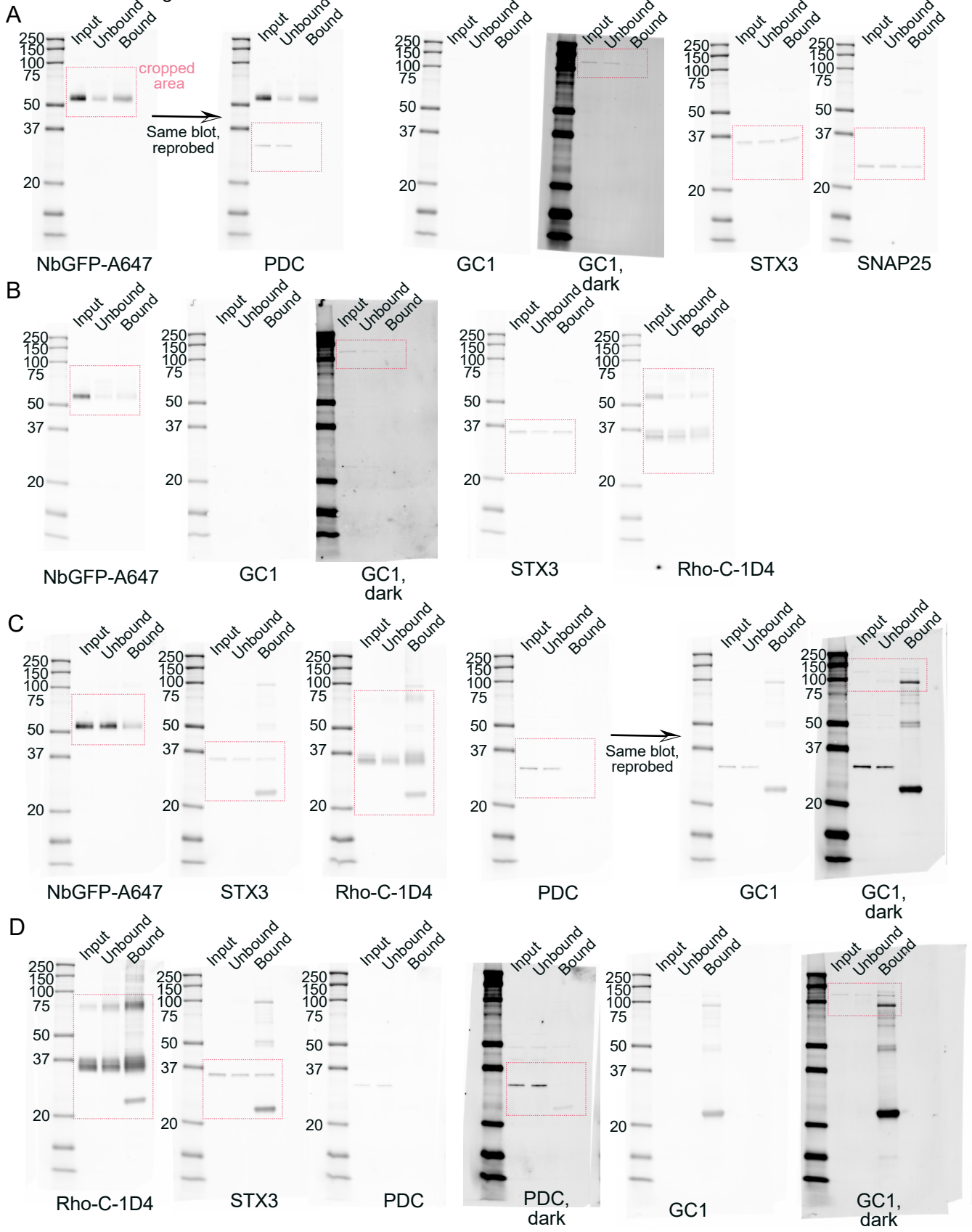

Supplemental Data for Figure S1

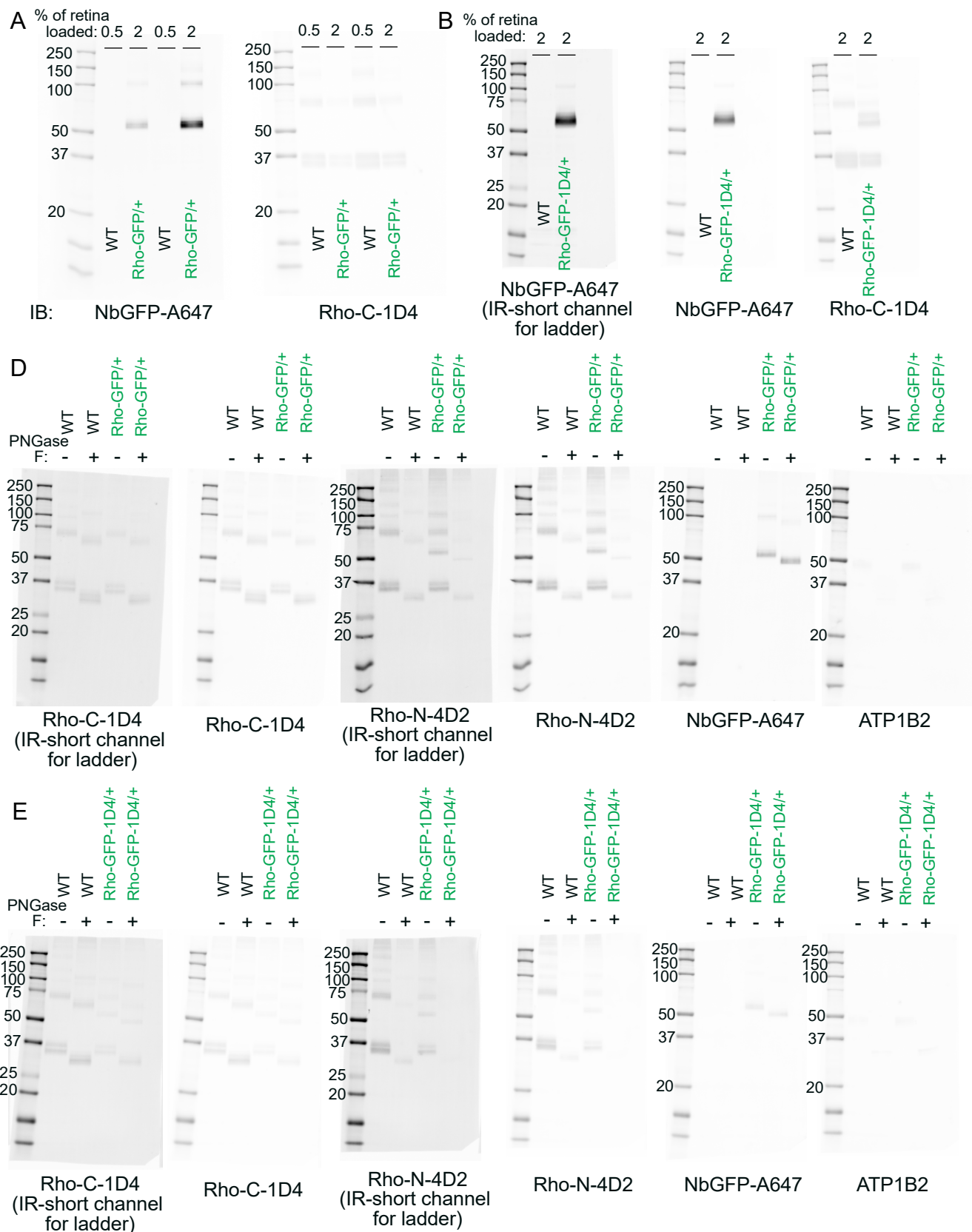

Supplement: S1 Raw Images — (PDF) [file pbio.3002467.s007.pdf]
