# Supplementary material for: Super-resolution mapping in rod photoreceptors identifies rhodopsin trafficking through the inner segment plasma membrane as an essential subcellular pathway
Source: PLoS Biol. 2024 Jan 8;22(1):e3002467. doi: 10.1371/journal.pbio.3002467 (PMC10773939; doi:10.1371/journal.pbio.3002467)
Supplement: Attachment — Submitted filename: ResponseToReviewers.docx [file pbio.3002467.s008.docx]

**Response to Reviewers**

For the submitted manuscript: “Mapping rhodopsin trafficking in rod photoreceptors with quantitative super-resolution microscopy” (PBIOLOGY-D-23-01212R1)

Authors:

Kristen N. Haggerty, Shannon C. Eshelman, Lauren A. Sexton, Emmanuel Frimpong, Leah M. Rogers, Melina A. Agosto, **Michael A. Robichaux***

(*****corresponding author, michael.robichaux@hsc.wvu.edu)

Author Response Summary

We are thankful and appreciative for the reviewers’ comments on our manuscript. We were delighted to read the positive reports that highlighted the quality of our imaging data, the rigor of our experiments, and the quality of our writing and discussion. Constructive feedback from reviewers 1 and 2 were used to generate new surface labeling super-resolution data and a new Figure that we feel strongly support our conclusions and have improved our manuscript. We have also revised the manuscript text in accordance with the reviewers’ suggestions. We are especially thankful to the reviewers for detailed suggestions that improve the clarity of our writing. The revised manuscript includes our new figure and also addresses many of the concerns described by the reviewers with text additions, edits and clarifications. A point-by-point response to reviewer comments follows.

**Point-by-point responses are in blue.**

Reviewer #1:

Rhodopsin is synthesized in the inner segment and traffics to the outer segment in the highly compartmentalized photoreceptor cell. This process is important for the maintenance of photoreceptor health and function. The manuscript by Haggerty et al. employed super-resolution microscopy that revealed the localization of rhodopsin in mouse rod inner segments. The images showed localization of rhodopsin and molecules involved in rhodopsin trafficking in remarkable detail, adding to our understanding of this complicated process.

A major concern is that much of the work relies on the removal of the outer segment compartments in live tissue. This process could potentially produce artificial rhodopsin mislocalization. This concern is underscored by the common observation that rhodopsin tends to mislocalize in stressed, degenerating retina. Providing an experiment as well as thoughtful discussion to refute this possibility would greatly strengthen the manuscript.

To support our finding that Rho localizes to the IS plasma membrane and to address reviewer concerns about the 1) consequence of outer segment peeling in live tissue, and 2) concerns related to the Rho-GFP knockin mouse lines, we have performed new surface labeling experiments for super-resolution imaging. These new data are compiled into the new Figure 7 in the revised manuscript and are described in a new paragraph in the Results section (starting at line 373). With this new data we show Rho-N-4D2 surface labeling throughout the IS layer in (unpeeled) WT retinas, as well as in Rho-GFP/+ and Rho-GFP-1D4/+ retinas. This finding supports our model that Rho localizes and is trafficked throughout the IS plasma membrane. The surface localization of Rho-N-4D2 STORM data was quantified and statistically compared among genotypes using the same analysis tools that we developed for the STORM data in the original manuscript. The conclusion from the new statistical analysis is that the IS surface localization of Rho is not significantly different between WT, Rho-GFP/+ and Rho-GFP-1D4/+ genotypes. We have added reference to these surface localization results throughout the revised manuscript text.

The reviewer raises a fair point regarding uncertainty about potential artifacts introduced by our OS peeling method in unfixed tissue. While we cannot validate with certainty that the OS peeling does not cause some degree of damage and artificial rhodopsin (Rho) mislocalization, we have now emphasized in the Results sections that all inner segments (ISs) analyzed from IS-enriched tissue were stained with ciliary antibody markers of the connecting cilia/basal body (CC/BB, anti-centrin staining). This point was added as a new sentence in the Results of the revised manuscript (line 229), and is re-emphasized later in the Results (line 304). We have also added a new paragraph to the Discussion in the revised manuscript (start at line 529) outlining this point, including: 1) the consideration of OS peeling being a source of cellular damage, 2) our evidence that our IS protein localization findings are not caused by artifacts due to damage, and 3) an acknowledgement that we cannot completely eliminate the possibility of minor artifacts. We also provide some new data in the response to Reviewer 2 (see below) that provides additional evidence that OS peeling does cause substantial IS protein localization changes. Finally, we believe our new surface labeling data (Figure 7) support our Rho IS plasma membrane localization finding from our experiments in IS-enriched retinas.

Minor comments:

1. Some paragraphs in the introduction read like general reviews and can be condensed. How the content of these paragraphs directly relates to the question being addressed by the manuscript is often not clear. A concluding sentence at each paragraph linking the cited studies to questions being addressed by the current study would be helpful.

Based on the reviewer’s suggestion, we have added concluding sentences or phrases to the Introduction section of the revised manuscript (lines 91, 130), edited existing concluding sentences (line 107) and condensed the content of Introduction paragraphs 2 and 5.

2. Line 21: efficient long-term phototransduction. Consider rephrasing.

We have rephrased our wording for clarity (See line 28 of the revised manuscript).

3. Line 56: CC is a thin ciliary bridge that spans 1.1 um. It would be more clear to define the diameter as well as length.

We have added the CC diameter to this sentence in the revised manuscript (line 57 of the revision).

4. Line 174: Rho-C-1D4 labeling sentence is a repeat of line 170.

We have deleted the redundant sentence and slightly modified the wording of the subsequent sentence (line 169 of the revision).

5. What is the conclusion from Fig. 1F data? There is no rhodopsin in the inner segment plasma membrane? What is the interpretation between the conflicting results in Fig. 1 C, D and F?

We have clarified our description of these data in the Results (line 204) as well as in Figure 1 and the Figure 1 legend. Figure 1F is a SIM image from a Rho-GFP/+ retina that was immunolabeled with NbGFP-A647 (as in Fig. 1 E) but using twice the amount of nanobody. As in E, we were unable to immunolabel the IS in these retinas, which led to our conclusion that our immunolabeling reagents could not penetrate to the IS sufficiently in full retinas. Thus we proceeded to use the retinal peeling approach described in Figure 2.

6. Line 294: Fig. 4D, E should be Fig. 5D, E.

We have fixed this typo (line 292).

7. The relatively poor colocalization between rhodopsin and rab11a in the inner segment is somewhat surprising. Do the authors expect this low degree of colocalization? Caption for Fig. 9 should reflect this partial colocalization.

We were surprised by this low level of colocalization; however, we are unsure if this may reflect a transient interaction between Rho and Rab11a, or a different role for Rab11a altogether. We are currently investigating this question as part of a follow-up study. Based on the reviewer’s comment, we have updated the legend for the now Figure 10, and added a sentence to the Discussion about the Rho-Rab11a colocalization data (line 559 of the revision).

Reviewer #2: Haggarty et al

This study applies quantitative super-resolution microscopy (SIM and STORM) to uncover localization of rhodopsin in rod photoreceptors and determine details about rhodopsin trafficking in the mouse rod IS. The authors use WT and a hRho-GFP/+ knockin mouse model in which the mouse rhodopsin gene was replaced by the human gene encoding a hRho-GFP fusion protein. Key results are that in confocal images of Rho-GFP/+ and Rho-GFP-1D4/+ retinas, GFP fluorescence was confined to the OS layer. Using SIM, a faint GFP signal was visualized in the IS layer from both knockin mice. Fluorescence with a GFP-specific nanobody NbGFP-A647 was limited to the distal OS tips, and only a few rods had any Rho-GFP labeling in the proximal OS near the centrin-positive CC (Fig. 1 E) showing no detectable fluorescent signal in the IS (Fig. 1 F). This result changed when IS-enriched retinas from Rho-GFP/+ mice were used. In these retinas, NbGFP-A647 fluorescence was now observed in the rod CC, IS and ONL regions among the sparse remaining OSs in these sections (Fig. 2 E). Based on these results, the authors propose a model of rhodopsin post-Golgi vesicle trafficking where rhodopsin containing vesicles fuse with the IS plasma membrane and traffic to the periciliary ridge by 2D diffusion.

Critique

This is an interesting manuscript employing state of the art imaging techniques to analyze localization of mouse rhodopsin or its hRho-GFP fusion proteins. Experiments are carefully carried out and statistically evaluated. This reviewer is unsure what it all means for GPCR trafficking in general. Authors firmly believe that fluorescence emitted by hRho-GFP represents the location of WT rhodopsin. The reviewer thinks that the hRho-GFP is a mutant, not identical to WT rhodopsin, and that its location in the IS PM differs from that of rhodopsin in WT animals.

Further, OS peeling to generate IS-enriched retina may damage the IS, particularly the microtubule cytoskeleton and cytoplasmic dynein. This could be the reason why the authors cannot colocalize hRho-GFP and dynein. There is plenty of evidence that GPCR trafficking in neurons depends on molecular motors. Specifically rhodopsin strongly depends on dynein for delivery to the outer segment.

We provide a response to the critique of potential damage caused by OS peeling below.

In the hRho-GFP mouse lines, the C-terminal of human rhodopsin is fused to GFP and the mutant mimics the distribution of wild-type rhodopsin in hRho/+ heterozygotes (Chan et al., 2004). hRho/+ mice eventually degenerate, hRho-GFP acts as a recessive mutant. Presumably some aspect of the structure of the hRho-GFP is responsible for the degeneration.

In hRho-GFP homozygotes, recessive retinitis pigmentosa is much accelerated suggesting that the hRho-GFP dimer is less stable than the hRho-GFP/mouse rhodopsin heterodimer, and mouse rhodopsin stabilizes hRho-GFP. Importantly, hRho-GFP is a mutant rhodopsin and foreign in the mouse retina. hRho-GFP/+ expresses both native mouse Rho and mutant hRho-GFP which form mouse Rho and mutant hRho-GFP dimers and presumably traffic as dimers.

It is unclear why the fusion protein is unstable, or whether hRho-GFP's C-terminal CLS is partially blocked by the bulky GFP. It is unclear whether native mouse rhodopsin distribution in WT retina is identical to hRho-GFP/mouse rhodopsin distribution in the mutant. It is also unclear whether the amount of Rho-GFP is increased in IS-enriched rod IS, or only better visualized when OS are removed?

We recognize the issues addressed by the reviewer and are appreciative for their thoughts on the subject. As outlined in the MS, we included the Rho-GFP-1D4/+ knockin mouse to address the potential occlusion of the CLs/1D4 C-terminal sequence in the Rho-GFP line (revised manuscript line 173). We also recognize the uncertainty regarding the precise dimerization state of hRho-GFP with mouse WT Rho in our mice, although we found they do coimmunoprecipitate from Rho-GFP/+ IS-enriched retinal lysates (Fig. 8 C). On this point, we note that we were encouraged to find that both Rho-GFP and Rho-GFP-1D4 proteins were glycosylated (Fig. S1 D-E). Such a deglycosylation analysis had not been performed previously (to the best of our knowledge) on Rho-GFP in mouse tissue, and this was evidence that the fusions do not follow an alternative trafficking pathway.

Currently, we are unable to effectively measure whether the amount of Rho-GFP changes during OS peeling, but our new surface labeling experiments reported in Figure 7 of the revised manuscript confirms Rho and hRho-GFP IS plasma membrane localization in WT, Rho-GFP/+ and Rho-GFP-1D4/+ retinas. This is also evidence that OS peeling does not cause a dramatic mislocalization of Rho that would increase the amount in the IS. Finally, we believe that our new surface labeling data is helpful in demonstrating that hRho-GFP localization is similar to WT Rho localization at the IS plasma membrane.

The retinal peeling technique (Fig. 2A) to remove OS to generate IS-enriched retina samples is very interesting. Outer segments are successively removed in unfixed rods, where the retina slices were peeled 8 times using filter paper. The time needed for 8 peelings and the effects on the IS are unclear.

We have added to the Methods that the peeling technique (8 peelings) is an approximately 10 minute procedure (line 717).

Some CCs which are contiguous with the axoneme are likely pulled out, a procedure which must affect the microtubule cytoskeleton in the IS (compare 2F with 1F). The peeling technique could be called a mechanical photoreceptor degeneration technique with unknown consequences on IS metabolism. Removal of the basal body, functioning as microtubule organizing center, disrupts microtubule anchoring to the BB, a procedure that could inactivate cytoplasmic dynein with consequences on survival of rods. Inactivation of dynein could push vesicles to IS membrane after peeling is complete, increasing hRho-GFP at the IS membrane.

We thank the reviewer for their analysis of OS peeling method, and we have included a new paragraph to the Discussion (line 529) describing the possibility that the method could be damaging alongside our discussion about how our data indicate that if damage occurs, it is minor and would likely not cause gross mislocalization in the IS. When developing the method, we were concerned, like the reviewer, that the procedure would mechanically remove all the BBs from the IS layer, and so we were relieved to observe that this was not the case. Still, as outlined in the revised manuscript (lines 229 and 304), we only analyzed rod ISs that still had an intact CC/BB (based on centrin immunostaining) to avoid any damaged ISs.

Based on the reviewer’s comment, we also performed a new experiment to image rod cell tubulin with SIM to determine if the OS peeling noticeably disrupted IS microtubule organization. We used the immunolabeling Methods outlined in the manuscript to compare WT full retinas to WT IS-enriched retinas immunolabeled with anti-centrin-2 (BioLegend #698601, yellow), anti-STX3 (Proteintech #15556-1-AP, cyan) and anti-TUBB5B (Sigma # T7816, magenta). While the tubulin labeling was dense in the IS, and is likely staining microtubules and cytoplasmic tubulin, we did not see any gross tubulin disorganization in the IS-enriched retina. Notably there appears to be an antibody penetration issue for anti-TUBB5B. Because we are pursuing an analysis of the IS cytoskeletal network in future studies, we feel that these data fall out of the scope of the current Rho manuscript, and we include example images from this staining here only as a response-to-reviewer figure.

See figure below:

Similarly, we do not observe any detectable cytoplasmic dynein (based on anti-DYNC1H1 immunolabeling) localization differences in full vs. unpeeled retinas (compare Fig. 10 G to Fig. 10K).

In the current form, the manuscript is not ready for publication in PlOS Biology.

Specific points:

Abstract: "Thus, our results collectively establish a model of rhodopsin trafficking through the inner segment plasma membrane as an essential subcellular pathway in mouse rod photoreceptors." As most of the rod IS results are based on GFP fluorescence, the results establish a model for hRho-GFP trafficking, not necessarily for mouse rhodopsin.

Although, we have taken care to edit the text of our revised manuscript to clarify when we are describing hRho-GFP, we feel in the case of the abstract, that our original wording of this sentence is fair since we are indicating that our data are used to “model” rhodopsin trafficking. We hope that adding Rho-N-4D2 surface labeling in Figure 7 helps balance our rod IS results to not be so heavily based on Rho-GFP localization.

Line 109: "Rho-GFP fusion knockin mouse." Chan et al. used human rhodopsin to generate the mouse line. hRho-GFP should be used instead of Rho-GFP.

We have made this correction (revision line 106).

Line 137: "rhodopsin trafficking within the IS remains largely uncharacterized". The term rhodopsin transport yields nearly 2000 responses in PubMed. There are plenty of unknowns, but rhodopsin trafficking has been investigated to quite an extent.

We have adjusted the wording here to “incompletely characterized” (revision line 133).

Line 175: "Rho protein in the IS was undetectable with immunofluorescence." But in 1A, there are traces of Rho in the IS with 1D4, 4D2.

We thank the reviewer for pointing out this misstatement. We have changed the wording to “not reliably detectable” (revision line 169).

LIne 210: "Even when we doubled the amount of NbGFP-A647 used during labeling, the Rho-GFP-localized fluorescence was still predominantly found in distal OS tips of Rho-GFP/+ retinas with no detectable fluorescent signal in the IS (Fig. 1 F)." Therefore expression of hRho-GFP after OS peeling increases? This provides a hint that OS peeling damages the IS.

We have adjusted this sentence for clarity based on a comment from Reviewer 1. It now reads: “Even when Rho-GFP/+ retinas were stained with double the amount of NbGFP-A647 (Fig. 1 F), the Rho-GFP-localized fluorescence was still predominantly found in the distal OS tips of Rho-GFP/+ retinas with no detectable fluorescent signal in the IS, indicating that NbGFP-A647 could not penetrate into the IS.” (revision line 204). Based on the distal OS staining pattern of hRho-GFP in Fig 1E and 1F that matched the distal OS staining pattern of Rho-C-1D4 immunolabeling in WT retinas in Fig 1B, we stand by this interpretation of the data.

Line 231: "IS-enriched rod examples had preserved CC structure that were typically attached to a remaining OS." Apparently, some CCs are pulled out together with OS which must affect the microtubule cytoskeleton in the IS (compare 2F with 1F).

We have added a subsequent sentence confirming this point (revision line 229) but stating that we only analyzed rods with intact CC/BBs.

Line 241: "Rhodopsin is localized at the mouse rod inner segment plasma membrane. " Rhodopsin should be replaced by hRho-GFP.

Although we understand the reviewer’s point of view about distinguishing hRho-GFP from WT mouse Rho, we feel that this Results section title is appropriate since this section includes the results from Figure 5 where we show endogenous immunolabeling of WT mouse rhodopsin, which is localized at the mouse inner segment plasma membrane.

Line 280-283:" In IS-enriched WT retinas that were immunolabeled with Rho-C-1D4 antibody, the endogenous Rho localization pattern matched the Rho-GFP IS pattern, including localization at the IS plasma membrane and CC membrane, in addition to prominent bright puncta labeling in the myoid IS." This could be due to hRho-GFP/mouse Rho heterodimer formation.

We politely point out that the data referenced here are images from WT retina sections stained for anti-Rho-C-1D4 (Fig. 5 A) and not from Rho-GFP/+ retinas.

Line 449: "Together these results indicate that vesicular Golgi and post-Golgi trafficking organelles are targeted to the IS PM in hRho-GFP/+ mouse rods." I added hRho-GFP/+ .

Once again, we feel that the original wording is more appropriate, since this statement is in reference to data from Figure 10A-F (from the revised manuscript) in which only WT retina samples were used.

Line 475. "Although we observed partial co-localization between Rho-GFP and DYNC1H1 with STORM, the overall abundant and diffuse pattern of IS DYNC1H1 is likely why the interaction strength with Rho molecules was not distinguishably higher than random." This maybe due to Dynein being in the inactive state, also called Phi state. If the microtubule cytoskeleton is damaged by OS peeling, Dynein stops interacting with cargo. The general assumption is that GPCR trafficking in neurons depends on molecular motors, see PMID: 35835604; specifically dynein, see Nemet et al. 2014, Dahl et al, 2021.

We appreciate the reviewer’s suggestion and expertise here. Still, we feel that our interpretation of the data is fair and still supports Rho cytoplasmic transport via dynein. As mentioned before, new projects are underway to explore the rod IS cytoskeleton, which will include a more in-depth analysis of dynein transport mechanisms, but we feel these important questions are beyond the scope of this manuscript.

Line 491: "suggest that after Golgi processing, new Rho proteins are trafficked within vesicles to the IS plasma membrane as a target/destination membrane." This is close to the standard hypothesis, that rhodopsin containing vesicles are unloaded at the IS PM near the periciliary ridge. In the author's model, unloading would happen anywhere at the IS PM. This still could be an artifact of the hRho-GFP mouse line.

We again acknowledge the possibility of minor localization artifacts in our analysis. But we hope that our new surface labeling data in the new Figure 7 provide additional supportive evidence for our model that Rho is being delivered to the surface all along the IS PM in mouse rods.

Line 498: "Furthermore, dense rod IS plasma membrane 499 localization of Rho was previously reported in pig retina (Röhlich et al., 1989), in mouse and 500 cow rods (Jan and Revel, 1974), and in developing rat rods (prior to OS formation) (Nir et al., 501 1984)." In older publications, this may depend on the quality of antibodies and fluorescence microscopes.

We agree with the reviewer’s point here; although, the Röhlich et al., 1989 study used diaminobenzidine immunocytochemistry instead of fluorescence, and the two other studies used immunoelectron microscopy methods.

Line 547: "Internal Rho that was sporadically localized in the ellipsoid IS may correspond to newly-synthesized Rho in the rough endoplasmic reticulum (ER)." The RER surrounds nuclei, this is where rhodopsin is biosynthesized. ER present in the ellipsoid must be smooth ER that is not involved in rhodopsin biosynthesis.

We thank the reviewer for pointing out this misstatement. We have removed “rough” from this Discussion sentence (revision line 578). Although Rho in the ellipsoid could be in smooth ER as suggested, we have preliminary evidence from another project that RER extends into the ellipsoid in mouse rods. However, we acknowledge that we cannot make this distinction in the current manuscript.

Reviewer #3: The study by Haggerty et al. uses super-resolution microscopy to interrogate the subcellular localization of rhodopsin, the visual pigment in rod photoreceptors, in the mouse retina. The localization of rhodopsin and other known molecules is determined in rod inner segments specifically. Two super-resolution microscopic techniques are used: SIM and STORM. The authors present convincing images of high quality. The work is well-done, the manuscript easy to follow and the conclusions well-supported by the data. I think that globally, the study confirms what was already known about the presence of many of the molecules imaged here. However, there are also new findings, such as the colocalization of STX3 and rhodopsin in the IS plasma membrane. The authors did an excellent job at discussing their data with the published data. The results collectively establish the power of super-resolution microscopy to interrogate protein trafficking in photoreceptors. The data also supports a novel of rhodopsin trafficking through the inner segment plasma membrane.
