# Supplementary material for: Super-resolution mapping in rod photoreceptors identifies rhodopsin trafficking through the inner segment plasma membrane as an essential subcellular pathway
Source: PLoS Biol. 2024 Jan 8;22(1):e3002467. doi: 10.1371/journal.pbio.3002467 (PMC10773939; doi:10.1371/journal.pbio.3002467)
Supplement: Attachment — Submitted filename: 231128ResponseToReviewers2.docx [file pbio.3002467.s009.docx]

**Response to Reviewers 2**

For the submitted manuscript: “Mapping rhodopsin trafficking in rod photoreceptors with quantitative super-resolution microscopy” (PBIOLOGY-D-23-01212R2)

Authors:

Kristen N. Haggerty, Shannon C. Eshelman, Lauren A. Sexton, Emmanuel Frimpong, Leah M. Rogers, Melina A. Agosto, **Michael A. Robichaux***

(*****corresponding author, michael.robichaux@hsc.wvu.edu)

Author Response Summary

We are again thankful and appreciative for the reviewers’ additional minor comments on our revised manuscript. A point-by-point response to reviewer comments follows.

**Point-by-point responses are in blue.**

Reviewer #2:

Minor comments:

Line 54 " Rho, which is synthesized in the rod photoreceptor inner segment (IS)." This is not correct. Rho is synthesized at the RER surrounding rod nuclei in the ONL. ONL is not part of the IS. Rho mRNA is exported from nuclei , translated by ER-associated ribosomes and incorporated into the ER membrane. From there, the trafficking pathway starts.

We thank the reviewer for this clarification. We have removed “synthesized” from this sentence and modified the wording of this sentence to simply state that Rho is trafficked through the photoreceptor IS to the OS (revision line 54).

Line 229. Cilia should be connecting cilia

We have made this correction (revision line 228).

Line 541 "Our model for IS Rho trafficking in mouse rods (depicted in Figure 10)..". The model depicted in Figure 10 was deleted in R2. Please delete.

We thank the reviewer for pointing out this error. In the revision, the model diagram was moved to the supplement. We have corrected the figure label here (revision line 541).

Line 981-1001: Yellow arrows are not mentioned in the legend of Figure 1.

We thank the reviewer for identifying this omission. The missing word (“arrow”) was added back to the figure legend in the revised manuscript (revision line 995)

Line 1239 Incomplete reference

We have corrected this reference (revision line 1244).
